# Functional analysis of novel SNPs and mutations in human and mouse genomes

**DOI:** 10.1186/1471-2105-9-S12-S10

**Published:** 2008-12-12

**Authors:** Chuan-Kun Liu, Yan-Hau Chen, Cheng-Yang Tang, Shu-Chuan Chang, Yi-Jung Lin, Ming-Fang Tsai, Yuan-Tsong Chen, Adam Yao

**Affiliations:** 1 National Genotyping Center (NGC), Academia Sinica, Taipei, Taiwan 11529, R.O.C; 2 Institute of Biomedical Sciences (IBMS), Academia Sinica, Taipei, Taiwan 11529, R.O.C

## Abstract

**Background:**

With the flood of information generated by the new generation of sequencing technologies, more efficient bioinformatics tools are needed for in-depth impact analysis of novel genomic variations. FANS (Functional Analysis of Novel SNPs) was developed to streamline comprehensive but tedious functional analysis steps into a few clicks and to offer a carefully designed presentation of results so researchers can focus more on thinking instead of typing and calculating.

**Results:**

FANS  harnesses the power of public information databases and powerful tools from six well established websites to enhance the efficiency of analysis of novel variations. FANS can process any point change in any coding region or GT-AG splice site to provide a clear picture of the disease risk of a prioritized variation by classifying splicing and functional alterations into one of nine risk subtypes with five risk levels.

**Conclusion:**

FANS significantly simplifies the analysis operations to a four-step procedure while still covering all major areas of interest to researchers. FANS offers a convenient way to prioritize the variations and select the ones with most functional impact for validation. Additionally, the program offers a distinct improvement in efficiency over manual operations in our benchmark test.

## Background

As sequencing technologies continue to advance rapidly and the cost of large-scale genotyping and sequencing falls markedly as a result, there is an increasing need for more efficient bioinformatics tools with which to analyze the flood of information regarding novel variations in human and mouse genomes. For instance, when novel mutations are found in a cancer genome re-sequencing project or when novel SNPs are pinpointed in a genetic study, a bioinformatics tool that can efficiently analyze and prioritize these novel variations for downstream research will surely save a lot of time and labor. Currently, though many well developed web projects like SNP@Promoter [1], F-SNP [2], and Snap [3] provide rich information for functional SNP prediction and integrated SNP annotation, they can only process known SNPs rather than newly found ones. In the analysis of novel variations, the Genomic Mutation Consequence Calculator (GMCC) [4] does offer some help to researchers by allowing human mutation analysis to the synonymous/non-synonymous level. However, to perform more in-depth impact analysis in human and mouse genomes, researchers still need to use a suite of separate online software applications like Rescue-ESE [5] for candidate exonic splicing enhancers, Fas-ESS [6] for candidate exonic splicing silencers, SIFT [7] for effects on protein function, and PolyPhen [8] for structure and function alterations of a human protein. These analyses of functional consequences caused by novel variations require many tedious and error-prone manual steps. To alleviate this problem, FANS (Functional Analysis of Novel SNPs) as an extended application of FASTSNP [9] streamlines information retrieval and functional analysis for multiple novel SNPs as well as mutations, with versatile input options and a graphical presentation of results, and provides a great efficiency improvement over manual operations.

## Implementation

### Design

According to their location and the kind of genetic alteration they cause, DNA variations are divided into five groups: non-synonymous, synonymous, splice site, intronic, and untranslated. Variations classified into the first three groups are those most likely to have significant functional impact. The first group may substantially affect protein function [10]: a non-synonymous variation leads to an amino acid change or makes a premature stop codon. Variations of the second [11] and the third categories [12] can affect pre-mRNA splicing. For better prioritization, FANS nominates variations as one of five risk types, with nine risk subtypes and five risk levels (Table 1) [13,14]. Each analyzed variation is assigned one risk type and an accompanying risk level. The impact analysis offered by FANS includes examination of diminished ESE or ESS, altered protein function, protein domain abolition, and GT-AG splice site variation. A very important design feature of FANS is that the analysis is always based on the most up-to-date data retrieved from the source websites NCBI (National Center of Biotechnology Information) [15], Ensembl [16], UCSC BLAT [17], Rescue-ESE [5], Fas-ESS [6], and SIFT [7].

**Table 1:** Risk types and risk levels used for prioritization of variations

**Group**	**Risk type**	**Risk subtype**	**Possible functional effect**	**Risk level**
Non-synonymous	Non-sense		Causes premature stop codon and affects protein function	Very High

Non-synonymous	Mis-sense	Non-conservative change	Predicts to affect protein function	High
		
Non-synonymous		Protein domain abolished	Changes ESE, ESS and known protein domain and results in protein structure abolished	High
		
Non-synonymous		Splicing regulation	Changes ESE, ESS but does not change known protein domain and results in exon splicing regulation	Medium
		
Non-synonymous		Conservative change	Does not change ESE, ESS and known protein domain and results in analogous protein structure	Low

Synonymous	Splicing regulation	Splicing regulation (protein domain abolished)	Disrupts exon splicing regulation, make protein domain abolished	High
		
Synonymous		Splicing regulation	Disrupts exon splicing regulation, make the same protein domain	Medium

Synonymous	Sense		Does not change amino acid and disrupt exon splicing regulation	Very Low

Non-coding	Splice site		Alters GT-AG splice site	High

### Input

Users of FANS can submit a single variation or a batch query. Currently, FANS offers users the option to search two species, human and mouse. After choosing the species, users need to input a sequence in FASTA format or provide information for each variation including chromosome number, physical position, orientation, and allele change, either through input fields on the web page or by uploading a batch file. For a batch file, each variation occupies a line that must contain the all required information for that variation. An example of a batch file is given online to illustrate the data format accepted by FANS. As all gene information and prediction results are acquired through the Internet in real-time, the maximum allowed number of variations for each batch query is currently 100 to avoid overloading the source websites.

### Analysis flow

**Figure 1:**
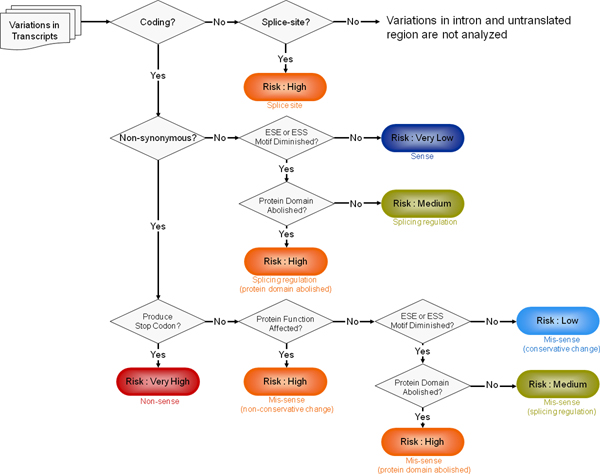
FANS prioritizing and analysis flow path.

Figure 1 outlines the analysis flow that is started when a query is submitted. For a sequence query, the FASTA format sequence is sent to UCSC BLAT [17] search. FANS automatically generates the required information about the novel variations from all the returned BLAT results, and users only need to choose a group of variations in one of the BLAT results for the next analysis stage. For each variation, FANS uses the submitted information to search NCBI Map Viewer to retrieve all transcripts of the gene covering the variation. If the variation falls in a non-coding region, it will be checked for its GT-AG splice site risk. Otherwise, the analysis will translate the coding region and then follow either a 'synonymous' or a 'non-synonymous' flow. A synonymous coding variation which affects amino acid sequence is first subjected to ESE and ESS analysis. The exonic DNA fragment of eleven nucleotides centered around the variation is extracted and transmitted to Rescue-ESE [5] and Fas-ESS [6] for ESE and ESS hexamers pattern matching. Any variation found to be covered by an ESE or ESS motif that is likely to diminish exon splicing is subsequently sent to protein domain abolition analysis. At this stage, FANS utilizes protein domain information from NCBI GenPept [18] to check protein domain abolition that may be caused by splicing regulation when the diminished exon splicing changes amino acid sequence in a protein domain.

A non-synonymous variation that produces a stop codon, resulting in very serious protein structure alteration, is categorized as risk type "Non-sense" with risk level "Very High". Other than the "Non-sense" type, FANS takes advantage of SIFT [7] to see if a particular amino acid replacement may affect protein function. Those variations predicted to affect protein function are categorized as "Mis-sense (non-conservative change)". If no significant functional impact is found despite the substitution of an amino acid, then ESE, ESS, and protein domain abolition analysis will be carried out.

### Output

**Figure 2:**
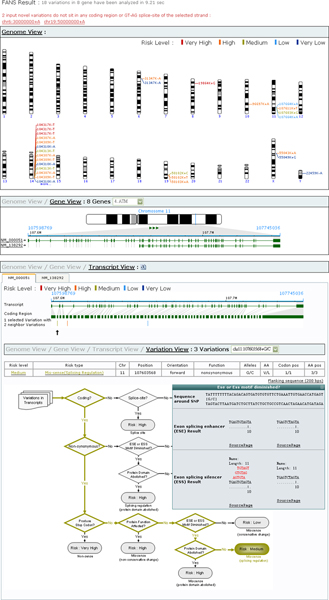
**The FANS results page**. The output of an analysis of eighteen variations in eight chromosomes.

By utilizing retrieved information and analyzed results from six websites, FANS efficiently prioritizes novel variations according to their risk levels in a few seconds with just a few clicks. The integrated results are divided into four parts for easy visualization: Genome View, Gene View, Transcript View, and Variation View (Figure 2).

The first screen, Genome View, shows an overview of the chromosome locations and the risk levels of the queried variations. The higher the risk level, the warmer the color that is used, with red color representing a very high risk level and blue a very low risk. Clicking on a variation label will move from Genome View to Gene View to display gene information of that variation. Gene View also displays the scale, location and all transcripts accommodating the selected variation. A gene selection list is provided for a user to choose the gene he/she is interested in. The transcript accession numbers from NCBI are printed on the left side of the transcript picture and transcript IDs from Ensembl are marked on the other side. Users can link to the source web pages by clicking each ID.

Next, in Transcript View, FANS offers separate transcript tabs for transcript selection (provided that a gene has more than one transcript). Gene structure comprising introns and exons is shown here. Extended exonic regions together with colored vertical lines are drawn for better illustration of variation locations. When the mouse is moved over a line, a pop-up window will show its risk level and risk type. In addition, an upwards-pointing arrow further distinguishes the position of the selected variation.

Finally, Variation View depicts the analysis flow path as well as the collected results of an analyzed variation. Selecting an interesting variation out of the variation list box will bring up its associated results and analysis flow path. FANS colors the flow path that an analysis has gone through according to the final risk level outcome. The highlighted path likewise provides corresponding description pop-up windows when pointed at with the mouse. Moreover, users can download not only all analyzed results in CSV format (by clicking the icon right of Transcript View) but also a 200 bps flanking sequence of any variation listed in Variation View.

### Development environment

The following software was used to construct FANS:

i. Red Hat Enterprise Linux 4 AS Update 6

ii. Eclipse 3.2.2

iii. Subversion 1.2.1

iv. Tomcat 5.5.20

v. RoboSuite 5.5 SP2

vi. Java SE Development Kit 1.5.0_11

vii. Struts Framework 1.3

viii. BioJava 1.5

Java is the core language for integration and data calculation from different websites and for the generation of the final results. BioJava, an open-source project, is used for amino acid translation. RoboSuite handles the submission and extraction of data from websites. All development processes were done on Eclipse and Subversion was adopted as our revision control system.

## Results

FANS automates analysis flow and simplifies operations so that researchers can spend more time on thinking instead of mouse clicking and dragging. Without FANS, the manual analysis of just a single variation requires a lot of tedious data extraction and transformation, which is compounded when there are many to analyze. Major processing steps include, for example, the retrieval of intronic and exonic information from every transcript of a gene covering the variation, the calculation of the position for translation if the variation occurs in a coding region, the extraction of an 11-mer sequence for ESE and ESS processing, the capture of protein domain information from NCBI GenPept flat file, the generation of an amino acid change for the SIFT program, and so on. In a manual operation benchmarking test, over an hour (3968 s) and 940 discrete steps were needed for a skilled person to operate all analytic processes over 10 variations in one gene (APOE). Two fifths of the operations were clicks for linking and submission, 170 steps were arithmetic, and others were copy/paste jobs. With FANS, the same analysis took only 118 s and 4 simple steps: selecting a query type, choosing a species, filling in variation information, and clicking the submit button, which dramatically reduced the time required and trimmed the returned results from forty-seven discrete pages to one single well-structured web page (Table 2).

**Table 2:** A benchmark comparison of analysis of 10 variations by hand and with FANS.

		**Manual**		**With FANS**
Number of Steps Needed	NCBI	350	940	4
			
	Ensembl	80		
			
	SIFT	100		
			
	Rescue-ESE	120		
			
	Fas-ESS	120		
			
	Calculation	170		

Time Taken		3968 s		118 s

Result Pages		47		1

## Conclusion

FANS is uniquely designed as a convenient way to perform in-depth integrated functional analysis of novel variations with always up-to-date data. With the application of next-generation sequencing platforms such as Roche GS FLX in genetic association studies or cancer research, many novel variations in patient samples will be unveiled. Under these circumstances, FANS offers a convenient way to prioritize the variations and select the ones with most functional impact for validation. In addition, more of the researchers' time and effort can be dedicated to productive thinking instead of error-prone manual operations. FANS significantly simplifies the analysis operations to a four-step procedure while still covering all major areas that researchers are interested in. Additionally, our benchmark tests showed that the operation time required for a sample analysis fell from more than one hour to less than two minutes. The sequence-based submission allows users to submit simply a human or a mouse genomic sequence containing targeted variations. The top-down design of views and easy data download plus many other intuitive features should make FANS quite user-friendly to researchers.

Our future development of this program will include database localization, an essential step to avoid overloading the websites, and one which should dramatically shorten processing times. The incorporation of more analytic tools such as PolyPhen [8] into our analysis flow is also an important goal in the further development of FANS.

## Availability and requirements

Project name: FANS

Project home page: 

Operation system(s): Platform independent

Programming language: Java

Other requirements: Standard web browser with JavaScript enabled

License: The tool is freely available to academic and non-academic users.

Any restrictions to use by non-academics: None

## List of abbreviations used

SNP: Single Nucleotide Polymorphism; ESE: Exonic Splicing Enhancer; ESS: Exonic Splicing Silencer; UCSC: University of California, Santa Cruz; CSV: Comma Separated Values.

## Competing interests

The authors declare that they have no competing interests.

## Authors' contributions

CKL designed the analysis flow and wrote the manuscript. CKL, YHC, CYT, and SCC implemented the program. CKL, YHC, YJL, MFT, and AY had substantial contribution to the user interface design. AY and YTC supervised the FANS project.
